# Radiomics-based fertility-sparing treatment in endometrial carcinoma: a review

**DOI:** 10.1186/s13244-023-01473-y

**Published:** 2023-07-19

**Authors:** Yuanjian Wang, Zhongshao Chen, Chang Liu, Ran Chu, Xiao Li, Mingbao Li, Dexin Yu, Xu Qiao, Beihua Kong, Kun Song

**Affiliations:** 1 Department of Obstetrics and Gynecology, Qilu Hospital of Shandong University, 107 Wenhuaxi Road, Jinan, 250012 Shandong Province China; 2 Gynecology Oncology Key Laboratory, Qilu Hospital of Shandong University, Jinan, Shandong China; 3 Department of Radiology, Qilu Hospital of Shandong University, Jinan, Shandong China; 4 School of Control Science and Engineering, Shandong University, Jinan, Shandong China

**Keywords:** Endometrial neoplasms, Radiomics, Fertility-sparing treatment

## Abstract

In recent years, with the increasing incidence of endometrial carcinoma in women of child-bearing age, to decision of whether to preserve patients’ fertility during treatment has become increasingly complex, presenting a formidable challenge for both physicians and patients. Non-fertility-sparing treatment can remove lesions more thoroughly than fertility-sparing treatment. However, patients will permanently lose their fertility. In contrast, fertility-sparing treatment can treat tumors without impairing fertility, but the risk of disease progression is high as compared with non-fertility-sparing treatment. Therefore, it is extremely important to accurately identify patients who are suitable for fertility-sparing treatments. The evaluation of prognostic factors, including myometrial invasion, the presence of lymph node metastases, and histopathological type, is vital for determining whether a patient can receive fertility-sparing treatment. As a non-invasive and quantitative approach, radiomics has the potential to assist radiologists and other clinicians in determining more precise judgments with regard to the above factors by extracting imaging features and establishing predictive models. In this review, we summarized currently available fertility-sparing strategies and reviewed the performance of radiomics in predicting risk factors associated with fertility-sparing treatment. This review aims to assist clinicians in identifying patients suitable for fertility-sparing treatment more accurately and comprehensively and informs more appropriate and rigorous treatment decisions for endometrial cancer patients of child-bearing age.

**Critical relevance statement**: Radiomics is a promising tool that may assist clinicians identify risk factors about fertility-sparing more accurately and comprehensively.

**Graphical abstract:**

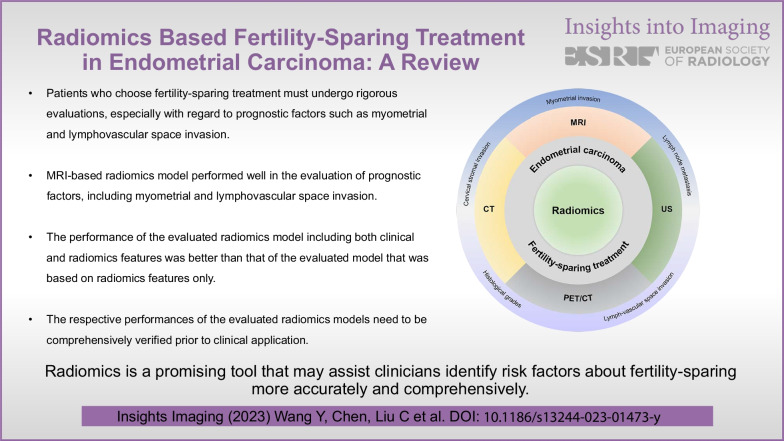

## Key points

1. Patients who choose fertility-sparing treatment must undergo rigorous evaluations, especially with regard to prognostic factors such as myometrial and lymphovascular space invasion.
2. We found that MRI-based radiomics model performed well in the evaluation of prognostic factors, including myometrial and lymphovascular space invasion.
3. We conclude that the performance of the evaluated radiomics model including both clinical and radiomics features was better than that of the evaluated model that was based on radiomics features only.
4. The respective performances of the evaluated radiomics models need to be comprehensively verified prior to clinical application.

## Introduction

Endometrial carcinoma (EC) is one of the most commonly occurring gynecological malignant tumors, and its morbidity ranks sixth among malignant tumors worldwide. According to cancer statistics published in 2020 [1], the expected number of new cases of uterine body malignancies (i.e., mainly EC) in the USA was 65,620 as of 2020, and EC ranks fourth among malignancies occurring in US women. EC usually occurs in postmenopausal women. However, in recent years, the incidence of EC in women of child-bearing age has been gradually increasing. Approximately 4% of EC patients are younger than 40 years of age [2]. Most of these patients strongly prefer preserving their fertility if possible. However, the risk of disease extension is inevitable in women who choose fertility-sparing treatment [3–6].

Patients presenting with different individual characteristics and histopathological features have different profiles in terms of viable treatments and treatment outcomes. Thus, the identification of early-stage patients and the assessment of prognostic factors, including myometrial invasion (MI), lymphovascular space invasion (LVSI), lymph node metastasis (LNM), and cervical stromal invasion (CSI), are critical for evaluating the appropriateness of fertility-sparing treatment [7–9]. As the current most commonly implemented preoperative assessment method, imaging can be used to preliminarily evaluate the size and location of lesions. However, some tiny lesions, such as early MI, are difficult to identify using imaging [9]. In addition, a large number of texture features that are potentially beneficial to diagnosis and staging are ignored [10].

As an emerging technology, radiomics can extract image features that cannot be identified by the human eye from imaging. Through processing, image features can be transformed into computer data. Based on these data or combining these data with clinical information, such as clinical features and pathology, it will be possible to develop more valuable diagnosis and prognosis models, which will guide clinicians to make better decision [11].

**Fig. 1:**
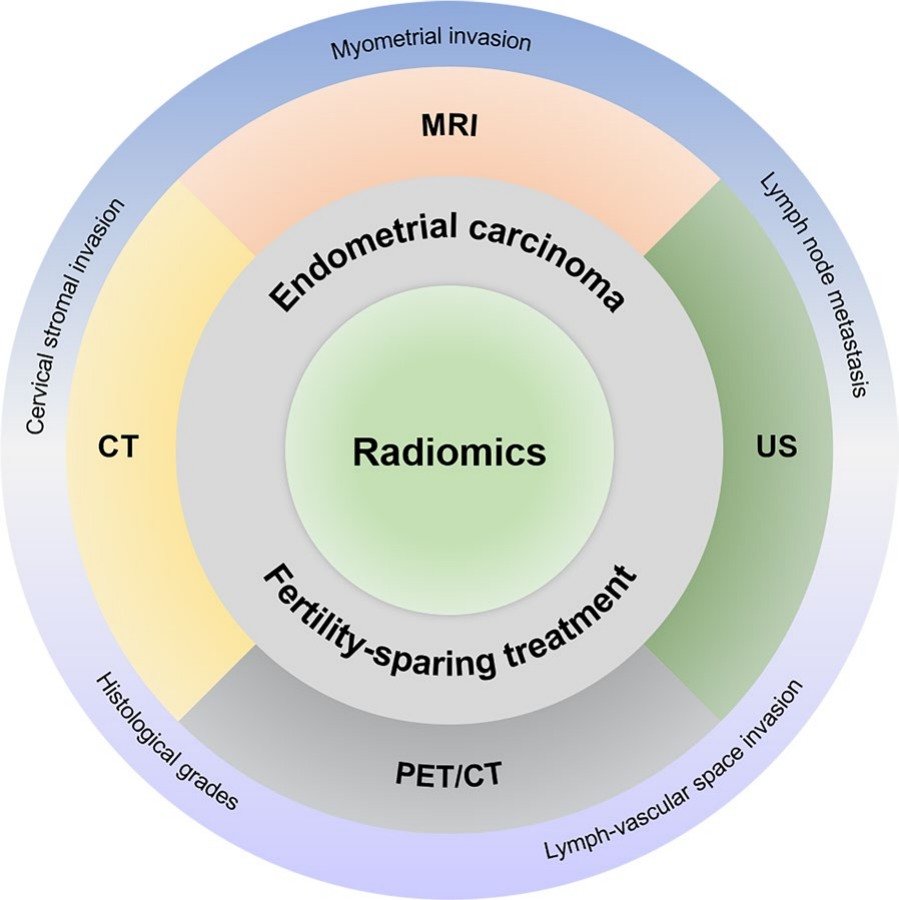
The application of radiomics in fertility-sparing treatment of endometrial carcinoma. MRI = magnetic resonance imaging. US = ultrasound. PET/CT = positron emission tomography/computed tomography. CT = computed tomography

This review summarizes currently available fertility-sparing strategies and the performance of radiomics models based on magnetic resonance imaging (MRI), ultrasound (US), computed tomography (CT), and positron emission tomography combined with CT (PET/CT) in terms of elucidating predictive prognostic factors associated with fertility-sparing treatments for EC (Fig. 1), including MI, LVSI, and LNM. We aimed to help clinicians determine better treatment choices for EC patients of child-bearing age.

## Methods

A search strategy was developed and applied to PubMed, Web of Science, and Scopus. The following words were used for searching: ((endometrial OR endometrium) AND (neoplasm OR carcinoma OR cancer OR tumor) AND (radiomics OR texture)) OR ((endometrial OR endometrium) AND (neoplasm OR carcinoma OR cancer OR tumor) AND (fertility-sparing OR fertility preservation) AND (radiomics OR texture OR imaging)). The search date ends in August 2021. A total of 604 results were searched in three databases; after removing repetition, the articles that are associated with the evaluation of risk factors related to fertility-sparing were included.

## Fertility-sparing strategies

The selection of eligible patients (among those choosing fertility-sparing treatments) is highly rigorous. The indications recommended by the National Comprehensive Cancer Network [12], the European Society of Gynecological Oncology [13] guidelines, and the International Federation of Gynecology and Obstetrics Cancer 2018 Report guidelines [14] for fertility-sparing treatment are shown in Table 1.

**Table 1:** Indications recommended by the National Comprehensive Cancer Network 2021, the European Society of Gynecological Oncology guidelines 2021 and the International Federation of Gynecology and Obstetrics cancer report 2018 for fertility-sparing treatment

Guidelines	Histopathologic types and grades	MI	MRI/US^a^	Dilatation and curettage or endometrial biopsy^a^	Informed consent^b^	Close follow-up	Outcomes^c^
National Comprehensive Cancer Network 2021	Grade 1 endometrial adenocarcinoma	No	Yes	Yes	Yes	Yes	TH/BSO
European Society of Gynecological Oncology guidelines 2021	Grade 1 endometrioid carcinoma or atypical hyperplasia/endometrioid intra-epithelial neoplasia	No	Yes	Yes	Yes	Yes	TH/BSO
International Federation of Gynecology and Obstetrics cancer report 2018	Grade 1 endometrioid carcinoma	No	Yes	-	Yes	Yes	TH/BSO

Patients who meet the above conditions should exclude pregnancy and consult a reproductive specialist before treatment. Genetic counseling and genetic testing are necessary for appropriate patients

MI, myometrial invasion; MRI, magnetic resonance imaging; US, ultrasound; TH/BSO, total hysterectomy and bilateral salpingo-oophorectomy

^a^To evaluate the extension of disease

^b^Fertility-sparing treatment is a non-standard treatment, which must be known for patients

^c^TH/BSO is recommended in the end, whether the treatment is successful or not

**Fig. 2:**
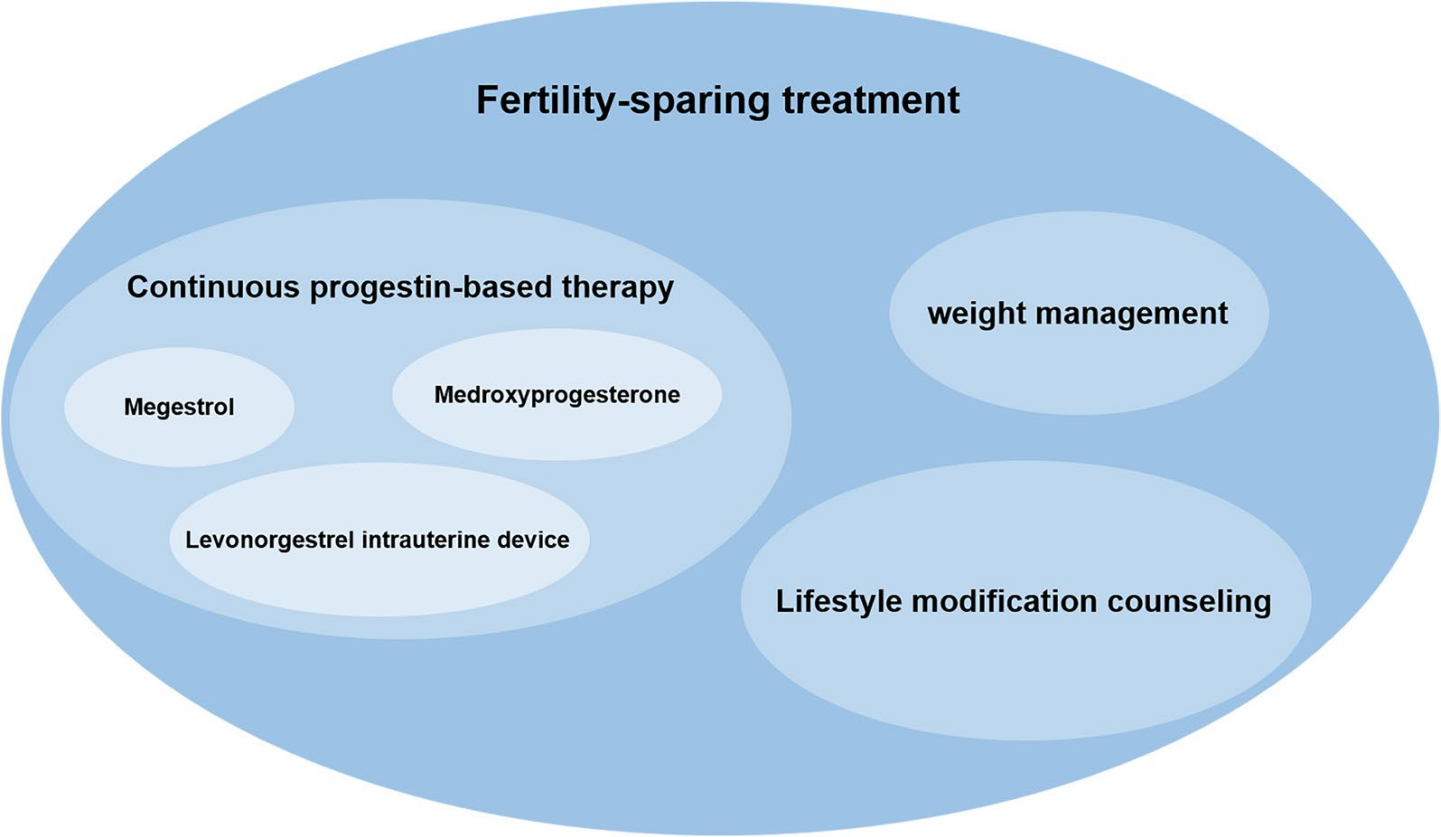
Current recommended fertility-sparing treatments of endometrial carcinoma

The standard treatment for women with EC who are of child-bearing age is total hysterectomy and bilateral salpingo-oophorectomy (TH/BSO). This is an effective method with a high five-year survival rate of 93% [2]. However, many of these women prefer fertility-sparing treatment due to the permanent loss of fertility caused by TH/BSO. Recommended fertility-sparing treatments are depicted in Fig. 2.

The most commonly prescribed therapeutic schedule is a standard regimen of high-dose oral medroxyprogesterone. The levonorgestrel intrauterine device can replace oral progesterone in women with complicated atypical hyperplasia [9]. In addition, there seems to be no correlation between diabetes and the outcomes of fertility-sparing treatment in women with atypical hyperplasia/endometrioid intra-epithelial neoplasia or early EC [15]. In contrast, the use of metformin may be associated with an increase in overall survival and a decrease in the risk of recurrence [16].

**Fig. 3:**
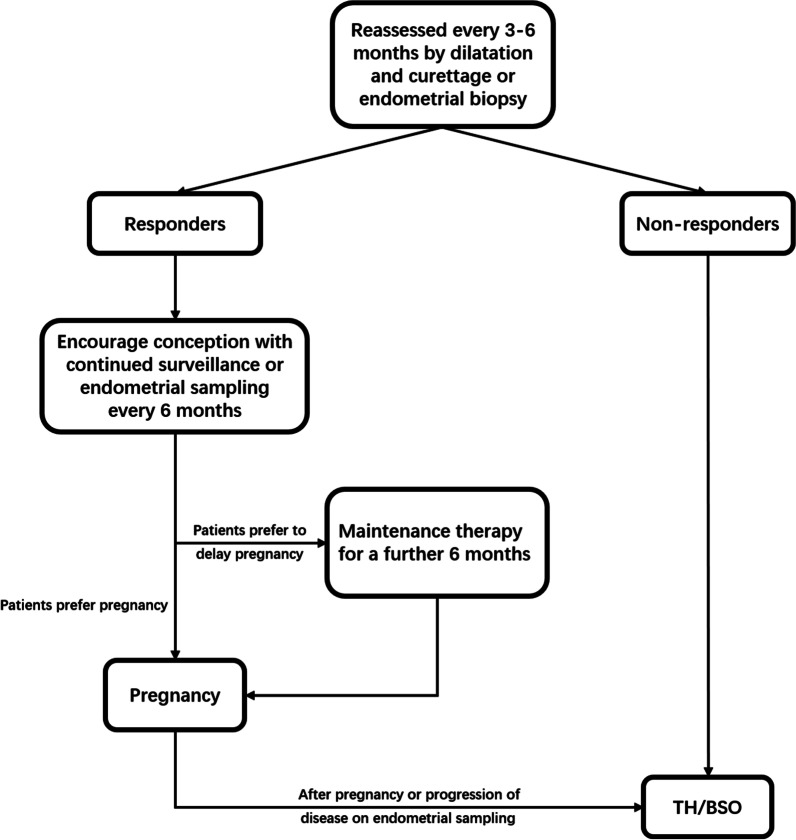
Flowchart of surveillance after fertility-sparing treatment. Responders: Complete response by 6 months. Non-responders: endometrial carcinoma present at 6–12 months. TH/BSO = Total hysterectomy and bilateral salpingo-oophorectomy

In addition to selecting appropriate treatments, close follow-up is an important factor in ensuring the safety and efficacy of fertility-sparing treatments. The surveillance strategies recommended by the National Comprehensive Cancer Network are shown in Fig. 3.

Moreover, the European Society for Medical Oncology has demonstrated that progesterone receptor status can reliably predict therapeutic response in EC. However, this indicator is not recommended as a routine test, because 50% of progesterone receptor-negative patients have been shown to respond to therapy [2].

## Imaging in fertility-sparing treatment

Currently, imaging (especially MRI and US) is one of the most commonly utilized methods for the preoperative assessment of EC [17–19]. Most clinical guidelines suggest the use of MRI to determine the extent of the lesions [20].

MRI with good resolution of soft tissues can distinguish endometrial lesions and myometrial signals to clearly show the range of lesions through contrast dynamic enhancement scanning. In recent years, MRI has been used to describe the local extent and expansion of tumors in various studies [21–23].

Moreover, US conducted by imaging experts has a nonnegligible role in evaluating the extent of EC in the pelvis and abdominal cavity. As a non-invasive, cheap, and convenient imaging method, US is appropriate for all patients. Epstein et al. revealed that ultrasonic and Doppler characteristics can be used for risk stratification in EC, and this finding was validated by a subsequent prospective study [19].

In contrast, CT is unreliable for predicting EC stage. This is because the contrast between the tumor and the myometrium is difficult to identify, thus rendering the tumor invisible in CT imaging. Thus, CT cannot be used to assess the depth of MI and CSI [24]. Due to good multi-plane spatial resolution, CT is mostly used for the preoperative evaluation of LNM and distant metastases. By analyzing CT images from 39 patients, Rizzo et al. concluded that dual-energy CT can overcome the limitations of traditional CT to distinguish lesions from normal tissue, thus providing a potential method for assessing the depth of MI in EC [24].

Additionally, PET/CT has been proven to be equal in performance as compared with MRI in terms of predicting MI [25]. However, the high associated cost of this methodology means that it cannot be considered a routine test.

Imaging has been widely used in clinical practice. However, the identification of tiny lesions is difficult in practice. In addition, it is difficult for radiologists and clinicians to acquire all imaging information associated with lesions through simple visual evaluations, and the differences between different imaging readers cannot be well controlled. Radiomics has the potential to overcome these problems.

## Radiomics

As a non-invasive approach, radiomics can extract quantitative and repeatable image features for analysis. Radiomics analysis includes the following six steps.

### Image acquisition

The acquisition of high-quality images is the foundation of radiomics, which has a meaningful impact on effective analyses. The uniformity of imaging machines and protocols should be ensured. However, if this is not possible, biases should be corrected via previously described protocols [11, 26].

### Image segmentation

The segmentation of images is critical for radiomics analysis. The region used in the subsequent analysis is obtained through image segmentation. For tumor analysis, the focus was the pixels and voxels in target area, which can be either two-dimensional or three-dimensional region. The two-dimensional region usually was the axial section where the maximum dimension of tumor is located and the three-dimensional region usually was the tumor itself. The segmentation method includes manual, semi-automatic, and automatic segmentation. Manual segmentation by an imaging expert is the gold standard for image segmentation [11, 26–30].

### Data preparation

Some key elements, including imaging methods, imaging protocols, and segmentation protocols, must be well defined through data preparation, which has a meaningful impact on model creation [11].

### Feature extraction

Radiomics features are defined as quantitative data extracted from imaging. Radiomic features are divided into four classes: first-order statistics, shape, texture features, and features obtained by wavelet transformation of relevant image sections. First-order statistics features can describe the distributions of voxel intensities. The shape features are associated with the shape of the volume. Texture features can reflect heterogeneity within tumors, including many gray-associated features. Wavelet features calculate the intensity and texture features from the wavelet decompositions of the original image [11, 27].

### Feature selection

Relevant features should be selected from among many radiomics features, and alternative features should be removed or transformed. This process is termed dimensionality reduction [29–31].

### Modeling

Establishing a model that can precisely predict the classification or prognosis of a disease is the primary goal of radiomics. There are many methods for establishing a radiomics model. Deep learning is the preferred method when the sample size is sufficiently large [11, 26, 29].

## Radiomics in fertility-sparing treatment

The performance of different radiomics models in predicting prognostic factors associated with fertility-sparing treatment is shown in Tables 2, 3, and 4.

**Table 2:** The performance of MRI-based radiomics in predicting DMI and LVSI

Factors and reference	Model and dataset type	Sensitivity	Specificity	Accuracy	AUC
DMI					
Stanzione et al					
	Model^R^				
	Training set	0.71 (10/14)	0.93 (27/29)	0.86 (37/43)	0.92
	Test set	0.67 (2/3)	1.00 (8/8)	0.91 (10/11)	0.94
Ueno et al					
	Model^R^				
	Only one set	0.79 (46/58)	0.82 (65/79)	0.81 (111/137)	0.84
Ytre-Hauge et al					
	Model^R^				
	Only one set	0.70 (53/76)	0.84 (83/99)	0.78 (136/175)	0.81
Kristine et al					
	Model^WT^				
	Training set	NA	NA	NA	0.84
	Test set	NA	NA	NA	0.76
	Model^SS^				
	Training set	NA	NA	NA	0.85
	Test set	NA	NA	NA	0.77
Yan et al					
	Model^CR^				
	Training set	1.00 (NA)	0.83 (NA)	0.87 (NA)	0.96
	Test set	0.72 (NA)	0.90 (NA)	0.87 (NA)	0.88
Zhu et al					
	Model^R^				
	Training set	0.95 (NA)	0.93 (NA)	0.94 (NA)	0.93
	Test set	0.95 (NA)	0.93 (NA)	0.94 (NA)	0.92
Alejandro et al					
	Model^R^				
	Only one set	0.81 (NA)	0.93 (NA)	0.86 (NA)	0.87
LVSI					
Ueno et al					
	Model^R^				
	Only one set	0.81 (55/68)	0.72 (50/69)	0.77 (105/137)	0.80
Luo et al					
	Model^R^				
	Training set	0.83 (NA)	0.73 (NA)	NA	0.82
	Test set	0.78 (NA)	0.79 (NA)	NA	0.81
Long et al					
	Model^R^				
	Training set	0.89 (32/36)	0.58 (59/102)	0.66 (91/138)	0.70
	Test set	0.86 (12/14)	0.63 (20/32)	0.70 (32/46)	0.75
	Model^CVF^				
	Training set	0.92 (33/36)	0.96 (98/102)	0.95 (131/138)	0.93
	Test set	0.93 (13/14)	0.63 (20/32)	0.72 (33/46)	0.81
Bereby-Kahane et al					
	Model^R^				
	Only one set	0.70 (19/27)	0.59 (27/46)	0.63 (46/73)	0.59

MRI, magnetic resonance imaging; AUC, area under curve; DMI, deep myometrial invasion; LVSI, lymph-vascular space invasion

Model^R^: Model constructed by radiomics features. Model^CVF^: Model constructed by radiomics and computer vision features. Model^CR^: Models constructed by clinical and radiomics features. Model^WT^: Model constructed by whole-tumor radiomics features. Model^SS^: Model constructed by single-slice radiomics features

**Table 3:** The performance of MRI-based radiomics in predicting LNM, CSI and histological grades

Factors and reference	Model and dataset type	Sensitivity	Specificity	Accuracy	AUC
LNM					
Ytre-Hauge et al					
	Model^R^				
	Only one set	0.68 (13/19)	0.73 (99/135)	0.73 (112/154)	0.73
Yan et al					
	Model^R^				
	Test set 1	NA	NA	0.80 (291/351)	0.91
	Test set 2	NA	NA	0.89 (240/271)	0.89
Xu et al					
	Model^R^				
	Training set	NA	NA	NA	0.79
	Test set	NA	NA	NA	0.75
	Model^C^				
	Training set	NA	NA	NA	0.87
	Test set	NA	NA	NA	0.83
	Model^CR1^				
	Training set	NA	NA	NA	0.89
	Test set	NA	NA	NA	0.88
	Model^CR2^				
	Training set	NA	NA	NA	0.84
	Test set	NA	NA	NA	0.82
Kristine et al					
	Model^WT^				
	Training set	NA	NA	NA	0.73
	Test set	NA	NA	NA	0.72
	Model^SS^				
	Training set	NA	NA	NA	0.83
	Test set	NA	NA	NA	0.56
CSI					
Ytre-Hauge et al					
	Model^R^				
	Only one set	0.53 (17/32)	0.78 (114/146)	0.74 (131/178)	0.64
Histological grades					
Ytre-Hauge et al					
	Model^R^				
	Only one set	NA	NA	NA	0.66
Zheng et al					
	Model^R^				
	Training set	0.72 (NA)	0.86 (NA)	0.79 (NA)	0.87
	Test set	0.77 (NA)	0.90 (NA)	0.83 (NA)	0.89
	Model^ADC^				
	Training set	0.61 (NA)	0.74 (NA)	0.68 (NA)	0.72
	Test set	0.43 (NA)	0.79 (NA)	0.62 (NA)	0.62
	Model^M^				
	Training set	0.89 (NA)	0.82 (NA)	0.85 (NA)	0.93
	Test set	0.92 (NA)	0.79 (NA)	0.85 (NA)	0.92

LNM, lymph node metastasis; CSI, cervical stromal invasion; AUC, area under curve

Model^R^: Model constructed by radiomics features. Model^C^: Model constructed by clinical features. Model^CR1^, Model^CR2^, Model^M^: Models constructed by clinical and radiomics features. Model^ADC^: Model constructed by apparent diffusion coefficient value. Model^WT^: Model constructed by whole-tumor radiomics features. Model^SS^: Model constructed by single-slice radiomics features

**Table 4:** The performance of US, CT and PET/CT in predicting DMI, LNM and CSI

Factors and reference	Model and dataset type	Sensitivity	Specificity	Accuracy	AUC
*US*					
DMI					
Alcazar et al					
	Van Holsbeke’s subjective model				
	Only one set	0.80 (33/41)	0.90 (103/114)	0.88 (136/155)	NA
*CT*					
DMI					
Ytre-Hauge et al					
	Model^R^				
	Only one set	NA	NA	NA	0.71
LNM					
Ytre-Hauge et al					
	Model^R^				
	Only one set	NA	NA	NA	0.69
CSI					
Ytre-Hauge et al					
	Model^R^				
	Only one set	NA	NA	NA	0.67
*DECT*					
DMI					
Rizzo et al					
	NA				
	Only one set	1.00 (13/13)	0.91 (20/22)	0.94 (33/35)	NA
*PET/CT*					
LNM					
Crivellaro et al					
	NA				
	Training set	NA	NA	NA	0.77
	Test set	0.43 (6/14)	0.93 (13/14)	0.68 (19/28)	NA
Elisabetta et al					
	Model^R^				
	Training set	0.75 (NA)	0.81 (NA)	NA	NA
	Test set	0.89 (NA)	0.80 (NA)	NA	NA

US, ultrasound; DECT, dual-energy computed tomography; PET/CT, positron emission tomography/computed tomography; AUC, area under curve; DMI, deep myometrial invasion; LNM, lymph node metastasis; CSI, cervical stromal invasion

Model^R^: Model constructed by radiomics features

### MRI

#### DMI

Deep myometrial invasion (DMI) is considered the most significant single morphological prognostic factor in patients with EC [32]. Ueno et al. [33] used a random forest model to assess DMI in 137 patients with EC. In terms of predicting DMI, prior research found that diagnostic accuracy (81.0%) and specificity (82.3%) were not statistically significantly different between the evaluated model and the evaluations of three radiologists with rich experience in gynecological imaging diagnostics, proving that this model can be considered a reliable auxiliary tool. However, their diagnostic accuracy for DMI was slightly lower than that of previous reports [34, 35]. This is because of the exclusion of 51 (24%) patients whose tumors may be too small to accurately determine contours. In a retrospective study, Kristine et al. [36] generated prediction models by whole-tumor and single-slice radiomics. For prediction of DMI, the area under the curve (AUC) of training and test cohorts was 0.84 and 0.76, respectively, in whole-tumor model. In single-slice model, the AUC was 0.85 and 0.77, respectively. Yan et al. [37] developed a nomogram by combining CA125, tumor size, and radiomics features to predict DMI. For radiologists, with the aid of nomogram, the performance of prediction of DMI was better than without nomogram. Zhu et al. [38] developed an MRI-based computerized method to predict DMI. This method only required uterus region rather than tumor region. Researchers defined a geometric feature called LS to evaluate the irregularity of the tissue structure triggered by EC. They formed a model called EPSVM by combining LS and texture features. The results revealed that compared with models in others studies, EPSVM had better performance in predicting DMI. Alejandro et al. [39] built four models using the Adaboost machine learning method to predict DMI. The best model included T2W texture, apparent diffusion coefficient (ADC) texture and statistical descriptors from ADC and semi-quantitative map images with the AUC of 0.87. A prospective cohort study designed by Ytre-Hauge et al. [40] included 180 patients with EC for magnetic resonance texture analysis. By delineating and analyzing the region of interest (ROI), 44 texture features were found to independently predict DMI. The most significant predictor of DMI had better accuracy (78.0%) and specificity (84.0%) as compared with traditional MRI readings. Although this study differs from the study conducted by Ueno et al. in terms of approach, these studies each indicate that MRI-based texture analysis has excellent potential for preoperative assessments of DMI in EC. Moreover, Stanzione et al. [32] established an MRI radiomics powered machine learning model to identify DMI in EC. The study included 54 patients, 17 of whom had DMI. With the help of the evaluated machine learning model, the accuracy of prediction increased from 82 to 100% (*p* = 0.48).

#### LVSI

The definition of LVSI is that tumor cells are lined by endothelial cells outside the immediate invasive border [41]. The model developed by Ueno et al. was found to be as accurate as a prior study using magnetic resonance volumetry in terms of predicting LVSI [42]. Moreover, in a retrospective study designed by Luo et al. [43], researchers discovered five radiomics features that predicted LVSI independently via least absolute shrinkage and selection operator (LASSO) regression analysis. The results demonstrated that the evaluated multiparametric MRI-based radiomics nomogram model predicted EC-associated LVSI effectively with an AUC of 0.807. This finding agrees with the conclusions of Ueno et al. However, Ueno et al. extracted only first-order statistical features. In contrast, the study conducted by Luo et al. extracted multiple comprehensive radiomics features and added clinical demographic features. Long et al. [44] extracted traditional radiomics features and computer vision features from preoperative T2-weighted and dynamic contrast-enhanced MRI and developed two models to predict LVSI. Model 1 was established according to traditional radiomics features, while Model 2 was established to assess improvements in performance after adding computer vision features to Model 1. The AUC for Model 1 was 0.79 and 0.75 in the training and test cohorts, respectively, whereas that for Model 2 was 0.93 and 0.81, respectively. These finding demonstrate that MRI-based computer vision nomograms have good performance in terms of prediction and applicability. Although the model established by Ueno et al. performed better than Model 1 in terms of predicting LVSI, the study conducted by Ueno et al. lacked an independent validation cohort. Additionally, research conducted by Bereby-Kahane et al. [45] suggested that the ability of MRI-based texture analysis to predict LVSI was limited. The reason for this finding may be that the enrolled sample size was small.

#### LNM

LNM is an important factor affecting EC prognoses [46]. Although the rate of LNM is low in the early stages of EC [47], the assessment of LNM is crucial for effective treatment decision-making and prognoses within EC. In a study conducted by Ytre-Hauge et al., five texture features were found to be statistically significant predictors of LNM via magnetic resonance texture analysis [40]. For prediction of LNM, the AUC of whole-tumor radiomics model established by Kristine et al. was 0.73 and 0.72 in training and test cohorts, respectively. And in single-slice model, the AUC was 0.83 and 0.56, respectively [36]. In addition, a multicenter study that established a radiomics model on preoperative MRI in 662 patients with EC helped radiologists predict LNM in EC using a random forest model [48]. The results showed that radiologists who were assisted by a radiomics model performed better than those without help in terms of predicting LNM, meaning that this model has satisfactory identification ability for detecting LNM in EC. However, under certain circumstances, even with positive prediction of LNM via the evaluated radiomics model, radiologists did not discover a LNM that was later verified by histopathology; this might be attributed to the uterus covering tiny lymph nodes or to the limited spatial resolution in MRI. Finally, Xu et al. [49] established four models showing good performance in LNM prediction based on the imaging and clinical characteristics of 200 patients with EC. The model integrating imaging features and clinical features (i.e., lymph node sizes and CA125) showed the best discrimination and accuracy, especially for normal-sized lymph nodes.

#### CSI

CSI is a risk factor for poor prognoses in EC. For early CSI, superficial lesions are often indistinguishable from the cervical mucosa on MRI, and the presence of chronic inflammation of the cervix often interferes with establishing diagnoses. This may in turn influence treatment decisions. If the presence of CSI can be accurately identified prior to surgery, this information would provide critical help for subsequent treatment decisions. However, according to a study conducted by Ytre-Hauge et al., there is no evidence that MRI-based texture analysis is conducive to the identification of CSI when compared with traditional MRI [40].

#### Histological grades

Histological grades, a vital prognostic factor, are important for choosing effective treatment strategies. Ytre-Hauge et al. reported that magnetic resonance texture analysis has the potential to predict histological grades in EC, as they found some features that were predictive high-risk histological subtypes [40]. Moreover, a retrospective study conducted by Zheng et al. [50] developed three models to predict histological grades in EC: Model^ADC^, which delineated the ROI manually in the apparent diffusion coefficient map; Model^R^, which segmented the tumor manually and subsequently extracted radiomics features; and Model^M^, which combined radiomics features with information on CA125 and body mass index (BMI). The performance of the Model^ADC^ was limited in the prediction of histological grades. In the training cohort, the AUC of Model^M^ was higher than that of Model^R^. However, the difference between Model^M^ and Model^R^ was not statistically significant in the test cohort. This may be attributed to the small sample size of the test cohort. In summary, the three evaluated models presented different levels of prediction. However, we concluded that Model^M^, which includes both radiomic and clinical features, was the most effective model by far.

#### Others

A large multicenter study conducted by Yan et al. [46] collected MRI data for 717 patients with EC. The ROI was manually drawn by a radiologist on each slice. After tumor segmentation, radiomics and clinical features were combined to establish a radiomics nomogram model and to evaluate the performance of this model in predicting high-risk EC. The results indicated that the radiomics nomogram had the highest AUC when compared with the combined radiomic and clinical features model. This finding and similar findings in the relevant literature demonstrate that radiomics has good diagnostic value for predicting high-risk EC.

### US

Women suspected of having EC must undergo two tests: transvaginal ultrasound and endometrial biopsy [51]. For the evaluation of endometrial thickness, transvaginal ultrasound is generally considered easier to calculate than transabdominal ultrasound. A recent meta-analysis showed that predicting DMI via Van Hoslbeke’s subjective model has acceptable sensitivity and specificity (80.5%, 90.3%), similar to the subjective judgment of radiologists assisted by transvaginal ultrasound (79.5%, 89.6%) [52]. Studies show that quantitative characteristics derived by US are closely associated with gestational age and respiratory disease in newborns and can also be used for the detection of thyroid and breast tumors [53]. For example, Liu et al. [54] extracted ultrasomics features from US images among 450 patients suffering from papillary thyroid carcinoma and used these features to predict preoperative LNM, thus showing the potential of this methodology for improving medical management and reducing overtreatment. Jin et al. [55] similarly extracted relevant ultrasomics features from ROI delineated by radiologists and attempted to predict LNM in early-stage cervical cancer using a non-invasive method. The results showed that ultrasomics features performed well in identifying LNM. However, to date, there have been few repeatable and stable studies that have obtained quantitative features from US imaging. Lesions of the endometrial junctional zone and ambiguous US findings, especially with respect to MI, can only be inferred from the irregular state of the endometrial junction [24]. In recent years, US has made great progress in the diagnosis of LNM and related diseases, showing its potential for the preoperative diagnosis of tumors and the effective prediction of risk factors. However, the feasibility and clinical applicability of ultrasomics remains to be verified due to the lack of research on ultrasomics to date.

### CT

Ytre-Hauge et al. [56] established model to predict DMI, CSI, and LNM by analyzing the tumor texture features from CT. The researchers identified 36 texture features in the statistical analyses. Entropy at filter level 6 (Entropy6) was the best predictor for DMI and CSI, and the AUC was 0.71 and 0.67, respectively. For prediction of LNM, Kurtosis5 was the top ranked feature with AUC of 0.69.

### PET/CT

PET/CT is an imaging method employing tracers, and the most commonly used tracer is fluorodeoxyglucose (FDG). FDG is taken up by cells that use glucose efficiently, such as tumor cells, and is subsequently detected by PET imaging. EC has a high associated rate of glucose metabolism and glycolysis, making it suitable for ^18^F-FDG PET/CT imaging. A prior retrospective study reported that volume density and irregular shape are the most strongly associated features for predicting LNM using PET/CT and that this method has high specificity for detecting LNM in EC [57]. Elisabetta et al. [58] extracted 75 radiomics features based on PET/CT imaging to predict LNM. The zone percentage of the gray-level size zone matrix (GLSZM ZP) was the feature with the lowest *p* value and highest AUC. The sensitivity and specificity in training cohort were 0.75 and 0.81, respectively. In test cohort, the sensitivity and specificity were 0.89 and 0.80, respectively.

## Prospects

This review summarizes progress in terms of radiomics-based fertility-sparing treatments in EC. In recent years, with the extension of research and multicenter clinical trials, the prediction performance of EC risk factors has become increasingly accurate. However, some issues and concerns still exist.

First, the existence of selection bias might have biased the results of this study. For example, when analyzing the performance of radiomics in predicting DMI, all enrolled patients underwent hysterectomy [32, 33]. To assess the accuracy of the forecast, it is necessary to accurately understand the presence or absence of risk factors. However, in the absence of surgery, this information is unclear. Paradoxically, this selection method ignores the influence of non-operative patients on prediction accuracy. A similar problem also exists when evaluating LVSI, LNM, and CSI [33, 43–45, 48, 49]. To solve this problem, patients without lymph node biopsy results were defined as negative when predicting LNM [46]. Obviously, this method is not sufficiently accurate. How to avoid bias produced by the inclusion of these patients should be discussed in future. If this problem can be solved, prediction accuracy will be improved concomitantly. Second, the delineation of target area is the most time-consuming step in radiomics workflow. The sample size, dimension of target area, and way of delineation will affect the time of undertaking radiomics. Manual segmentation is the gold standard, but it takes up too much time. A tool that can segment tumors effectively and automatically has the potential to save time and overcome inevitable inter-physician differences. This is a potential topic that merits comprehensive study in clinical research. Third, the influence of MRI scan thickness on feature extraction is unclear when determining the volume of interest (VOI) of tumors in whole-volume tumor analysis. Differences between whole-tumor derived features and single-slice derived features need to be compared more comprehensively in future research. Fourth, clinical features such as CA125 and BMI were added to the prediction model in some studies in order to improve prediction performance. However, to date, no study has added known risk factors, such as DMI, LNM, and LVSI, to prediction models in order to observe whether these factors influence each other. Fifth, most of the studies published to date were retrospective and their sample sizes were not large enough so as to be sufficiently powered. In future, multicenter prospective studies with large sample sizes are needed to test the respective performance of each evaluated prediction model. Sixth, the steps of radiomics analysis, including image segmentation and feature extraction, require external tools. Radiologists cannot conduct radiomics analyses directly on existing platforms for the purpose of image analysis and reporting. If radiomics analysis software can be integrated into existing imaging platforms, the convenience of implementing radiomics analyses will substantially improve. Seventh, ideally, a standardized image acquisition, imaging segmentation, and feature extraction process should be defined well in radiomics workflow. But in current situation, this is very difficult to achieve. In different center, the type of equipment, the protocols of imaging, and the version of software are usually different, which may influent the repeatability of radiomics features and models. The normalization of the data is a topic worth discussing in future. Eighth, at present, the application of radiomics is still in exploratory stage. The normalization and quality control of radiomics process and the repeatability of radiomics models are needed to be improved before radiomics is applied to clinical practice. With the popularity of interdiscipline, the cooperation of clinical doctors, radiologists, and computer technicians will promote the development of radiomics.

## Conclusions

We summarized the performance of radiomics in the evaluation of risk factors related to fertility-sparing. Radiomics is a promising tool that may assist clinicians identify risk factors about fertility-sparing more accurately and comprehensively. However, more research should be done before deploying radiomics into radiologists’ daily practice.

## Data Availability

Not applicable.

## Abbreviations

ADC: Apparent diffusion coefficient
AUC: Area under curve
CSI: Cervical stromal invasion
DMI: Deep myometrial invasion
EC: Endometrial carcinoma
LNM: Lymph node metastasis
LVSI: Lymphovascular space invasion
MI: Myometrial invasion
TH/BSO: Total hysterectomy, bilateral salpingo-oophorectomy
